# Multistakeholder Initiatives and Sustainability? A Governance Analysis using the German Initiative on Sustainable Cocoa (GISCO) as a Case Study

**DOI:** 10.1007/s00267-024-02050-9

**Published:** 2024-09-20

**Authors:** Daniel Schuster, Ivo Mossig

**Affiliations:** Institute of Geography, University of Bremen, Bremen, Germany

**Keywords:** Multistakeholder initiative (MSI), Network governance, Global economic relations, Cocoa and chocolate sector, Wicked problems

## Abstract

This paper examines the Multistakeholder initiative (MSI) “German Initiative on Sustainable Cocoa (GISCO)”. MSIs represent arenas in which heterogeneous actors from governments, businesses and civil society come together to achieve sustainability goals that they cannot achieve otherwise. The self-defined goals of GISCO are first, to improve the living conditions of cocoa farmers and their families; second, to conserve and protect natural resources and biodiversity; and third, to increase the share of sustainably produced cocoa. Although all stakeholder groups share these goals, they have different agendas and conflicting interests. Despite numerous case studies, no theoretical basis has been established on the functioning and success of negotiations in MSIs. Therefore, the question arises as to how the governance of an MSI can be captured empirically to explain (un)achieved outcomes of the collaboration. The contribution of this paper is the development of a theoretical framework and its application to the case study. Minutes of 84 meetings and 18 qualitative expert interviews were analyzed by social network analysis and qualitative content analysis using MaxQDA to identify (a) influential actors, (b) collaboration structures and (c) processes as well as (d) topics discussed to explain (not) achieved outcomes regarding the self-defined goals. The results provide detailed insight into the governance of an MSI. The MSI helps members to extend their individual networks and to learn from each other, but quickly reaches its limits in achieving the self-imposed common goals. One reason for this is the lack of representation of actors from the Global South, despite addressing environmental and living conditions there in two out of the three GISCO goals. Furthermore, it is shown that the structures and processes of decision-making within the MSI are designed in such a way that a lack of hierarchical directives, sanctions and other decision-making mechanisms makes negotiation-based compromises difficult. Consequently, the power of each stakeholder group to use their veto right to delay or prevent the changes required to achieve common goals cannot be overcome.

## Introduction and Research Question

Sustainability (Bundlandt 1987, Poveda and Lipsett 2011) is a frequently used term today that can be applied to almost all fields. Agri-food systems are also facing numerous sustainability problems. These include land use conflicts caused, for example, by land-grabbing (Lay and Nolte 2018) or by the growth of cities and the associated conversion of agricultural land, which is accompanied by the marginalization of farmers (Follmann et al. 2021) or the loss of biodiversity and the mitigation of greenhouse gases (Vandermeer et al. 1998: 2). In addition, poor and exploitative working conditions, especially for women and migrants (Corrado and Caruso 2022, Samonova 2022), abusive child labor (McKinney 2015) as well as living conditions and persistent poverty of farmers (Vicol et al. 2019) have been criticized for a long time. The studies cited above generally point to globalized interdependencies and, in particular, the role of powerful corporations from the global North due to their financial strength and market dominance, which view farmland as an asset class (Ouma 2020) and subordinate or ignore social and ecological aspects.

This non-exhaustive list of existing challenges in agri-food systems is an example of so-called wicked problems. Underlying them are global contexts characterized by a high degree of scientific and social complexity (Dentoni and Bitzer 2015; Dentoni et al. 2018; Levin et al. 2012; Rittel and Webber 1973). Accordingly, solving wicked problems represents a difficult undertaking. Grimm (2019: 9) discusses the activities of globally operating companies that (co-)create such problems. Their involvement in solving them is subject to the problem that a corporate commitment to sustainable development is associated with economic disadvantages compared to non-committed competitors. The same is true at the intergovernmental level. National governments are apparently unwilling or unable to exercise a regulatory function (Mena and Palazzo 2012), which is why corresponding negative socioeconomic and environmental circumstances arise as a result of the underregulation of global economic interrelationships.

As a consequence, examples from the fields of landscape governance like biodiversity loss, climate change, food insecurity, and poverty at the landscape show that partnerships between governments, businesses and civil society organizations are gaining importance and influence (Abarzúa and Glückler 2023; Aké and Boiral 2023; Bäckstrand 2006; Dentoni and Peterson 2011; Nadvi 2008; Ros-Tonen et al. 2018). However, the phenomenon of governments, businesses and civil society organizations engaging in sustainable development is not limited to landscape governance. We can observe different topics and geographical scales in which stakeholder groups act together in a variety of ways. Examples include public-private partnerships as a highly political influenced instrument for private-sector investment in the expansion of public infrastructure (Tang et al. 2023) or for co-promoting green finance by the private sector and the State (Ozili 2023) as well as collaborations for public healthcare (Falcone and Sica 2023; Schwartz and Yen 2017). The example of the municipal waste management system in Italy (Falcone and De Rose 2020) illustrates that conflicts are interwoven with the actions of the groups of actors involved. The government’s authoritarian approach has led to civil society actors joining forces and collaborating more closely together. They are now a stronger counter-movement than before (Falcone et al. 2020).

Multistakeholder initiatives (MSI), which are used to achieve sustainability goals, offer a concrete way for different groups of actors to cooperate (Bush et al. 2015). MSIs are defined as “[…] formalized arrangements in which organizations from diverse sectors (private, public, and not-for-profit) commit to work together in mutually beneficial ways to accomplish goals that they otherwise could not achieve alone” (Sloan and Oliver 2013: 1837). The literature contains various narratives of MSI approaches (Siangulube 2024), but two forms of MSI appear particularly frequently: On the one hand, there are standard-setting MSIs such as the Forest Stewardship Council (FSC) (Garrelts and Flitner 2011) or the Roundtable on Sustainable Palm Oil (RSPO) (Schouten et al. 2012), in which actors negotiate the concrete development and application of product or service certifications. On the other hand, MSIs exist that correspond to the “continuous improvement model” (Buckley et al. 2019). The MSI analyzed in this paper corresponds to this type, in which members jointly develop principles and indicators to capture and achieve self-imposed sustainability goals that are negotiated by participating actors and stakeholder groups. This typically focuses on a sector’s entire set of relationships along the global value chain with the goal of achieving successive improvements (Buckley et al. 2019). Analyzing an MSI according to the continuous improvement model expands the current state of the art because negotiation between heterogeneous actors with divergent goals has been studied primarily only in standard-setting certification initiatives (de Bakker et al. 2019; Wijaya et al. 2018).

Critics point out that such a collaboration does not necessarily ensure constructive and solution-oriented cooperation (Gray and Purdy 2018). Moreover, due to voluntary participation within MSIs, decisions made generally do not have any legally binding effect and are therefore referred to as “soft law regulation” (Mena and Palazzo 2012). However, such a dialog offers the opportunity to meaning-fully complement regulations through legally non-binding collaboration. Additionally, given the complexity and urgency of wicked problems, there seems to be almost no alternative for the different groups of actors to engage in a communicative exchange. In an MSI, there is a certain cognitive closeness between actors due to a common objective (Schneiker and Joachim 2018), but they may also have conflicting interests and priorities that seem difficult to reconcile (Grabs and Ponte 2019; Søreide and Truex 2013; Wijaya et al. 2018). If and how conflicting interests can be negotiated in an effective way directly touches on the question of governance of such a collaboration (Vangen et al. 2015). This requires a perspective that shifts the focus to the inside of the collaboration itself. In such a perspective, MSIs are understood as an arena where heterogeneous actors meet. However, a common understanding about the governance of MSIs has not yet been established within interdisciplinary discourse (Bryson et al. 2015; de Bakker et al. 2019; Gazley 2017; Getha-Taylor et al. 2019; Glückler 2020; van Tulder et al. 2016). The central question of this paper is therefore: how can the governance of an MSI in global economic relations be captured empirically to explain (un)achieved outcomes of the collaboration?

To answer this question, we propose a theoretical framework for capturing and analyzing the governance of MSIs. We build this on work on the governance of interorganizational (corporate) networks (Glückler 2020; Glückler and Németh 2012; Klijn et al. 2010; Provan and Kenis 2008). We understand the concept of networks from a relational perspective, which contributes to a better understanding of social processes on the one hand and social relations on the other (Grabher 2006). Here, in addition to the individual position of the actors within the network, both the qualities of the individual relationships and the overall structure of the network are visible and can be analyzed.

The paper is structured as follows: In the following section “The Governance of MSIs”, the theoretical framework regarding the governance of MSIs is developed and implemented using the case study of a German MSI in the cocoa and chocolate sector, the German Initiative on Sustainable Cocoa (GISCO). The initiative is introduced (section “The “German Initiative on Sustainable Cocoa” Case Study”) and the methods of protocol analysis and guided expert interviews are outlined (section “Methodology”). This is followed by a presentation of the empirical results (section “Results”). The paper then ends with a concluding summary (section “Conclusion”).

## The Governance of MSIs

As a starting point for the conceptualization, Vangen et al. (2015: 1244) define the governance of cross-sectoral collaborations, which include MSIs: “The governance of a collaborative entity entails the design and use of a structure and processes that enable actors to direct, coordinate and allocate resources for the collaboration as a whole and to account for its activities.”

Three key elements of governance can be derived from this definition: (a) actors, (b) processes, and (c) structures. The word “use” highlights the understanding of governance as a phenomenon that is constituted by the actions and interactions of actors (Bryson et al. 2015). Consequently, addressing these elements must go beyond “rules on paper or institutional design” (Bartley 2022: 189). Accordingly, the literature on MSIs, collaboration governance, and network governance indicates negotiated (d) topics and outcomes as the fourth element (Clarke and MacDonald 2019; Edelenbos et al. 2010). In the following, the state of research on these four elements of network governance is elaborated in order to derive specific sub-questions that, in accordance with the overarching research question, lead to the empirical measurement of governance thereby explaining (un)achieved outcomes of the collaboration.

### Actors

Formally, all individuals and (parts of) organizations involved in the MSI constitute the group of actors (Berardo et al. 2020). The question is, which actors are involved, and which are not? Are there barriers to access or selection procedures that lead to an “invisibilization effect” (Bitzer and Marazzi 2021: 382)? This is understood as the exclusion of certain groups of actors and their demands, which puts MSIs at risk of overrepresenting the interests of multinational companies. Glückler and Németh (2012) and Stone et al. (2013) emphasize that participating actors are embedded in specific contexts. Participants in an MSI are thus confronted with or embedded in complex rules, norms, and conventions at the level of societal macrostructures in a variety of ways, which influence their respective positions and options for action in the negotiation processes (de Bakker et al. 2019).

Differences between actors arise from their motivation for engaging in an MSI. Gray and Purdy (2018: 27–34) distinguish between competence-, resource-, legitimacy- and society-oriented participation motives, which civil society organizations often weight completely differently than the participating companies. Voluntary membership is ultimately based on generating at least one of the aforementioned benefits that justifies the resources used. In this context, actors differ in the extent to which they prioritize their self-interests (Grabs and Ponte 2019). Imbalances can arise in an MSI because the voluntary nature means that there are hardly any possibilities for sanctions or that decisions can be enforced via hierarchical directives (Glückler and Németh 2012), as exclusions or threats of withdrawal endanger the stability of the collaboration. Consequently, the extent to which actors agree with the common goals of the MSI and weigh these against their own interests is a key factor for stability but also for effectiveness with regard to the MSI’s self-imposed goals. The heterogeneity of the actors regarding the goals is thus a relevant variable (Ansell et al. 2020).

Within an MSI, according to the basic idea of such associations, the actors meet on equal footing. The actors are equal with regard to their statutory rights, obligations and opportunities for shaping the organization. However, Vangen et al. (2015: 1246) point out that individual actors are the driving forces in leading and shaping collaboration. They define such actors as “[…] anyone with enough power and know-how to influence and enact the collaboration’s agenda.” They can act powerfully based on their individual personalities, for example, through skillful negotiation, selective use of veto power, and/or via their active engagement in the collaboration (Crosby and Bryson 2010; Czischke 2018). However, disparate engagement or strict division of labor among actors or groups of actors can be problematic. Using an MSI in the Indonesian cocoa sector as an example, Wijaya et al. (2018) found that actor groups divided tasks according to competencies, characteristics, and resources, and thus contributed to the MSI’s work in different ways. This resulted in increasing tensions arising from the fragmentation of responsibilities and contributions.

### Structure

Vangen et al. (2015: 1246) define structure as the formal interconnections of all partners involved for the purpose of the collaboration. In view of the heterogeneous interests of the actors involved, decision-making structures of an MSI are of particular importance in the tension between inclusion and efficiency (Ansell et al. 2020; Provan and Kenis 2008; Stone et al. 2013). The formal design of structures can be represented in an organizational chart of the MSI. For example, elections (e.g., to the Executive Board) provide actors with specific decision-making authority associated with the offices. Following Provan and Kenis (2008), the structures of such collaborative networks can take three ideal-typical forms, which differ from each other in the positioning of the formally legitimized actors: “shared-governance,” “lead-organization governed networks,” and networks with a coordinating “network administrative organization (NAO).” In the case of shared-governance and lead-organization governed networks, the network is led from within, either distributed among different members or by a leading actor who is assigned the leadership role based on certain characteristics (Provan and Kenis 2008). In contrast, in a NAO, the network is coordinated by specially dedicated committees and positions, usually consisting of a steering board and an executive network management. The use of a NAO lends itself to MSIs because complex tasks and a relatively large number of participants, some with divergent interests, need to be coordinated.

However, the structures of the network are not only shaped by the possibilities of influence formally defined in its constitution. Glückler and Németh (2012) point out the parallel existence of formal and informal possibilities of influence that actors have in networks. For example, Abarzúa and Glückler (2023) use the example of a Chilean MSI for water supply to demonstrate that the informally created network structure does not correspond to the formally planned structure. Conceptualizing MSIs as networks enables the use of social network analysis and the creation of network graphs to identify opportunities for influence from the network position (Carboni et al. 2017; Fliervoet et al. 2016; Ulibarri and Scott 2017).

The specific internal institutions of the network provide a link between the “structure” and “processes” elements of network governance. Institutions can be divided into formal and informal (North 1990), because while the former are based on sets of rules such as statutes, contracts, or regulations, the latter emerge in concrete practices and actions (Bathelt and Glückler 2018: 201-202). In general, internal institutions are part of the structures. However, they arise through processes that contribute structural development, so that structures and processes are interwoven. Especially at the beginning of a network formation, many processes are aimed at developing formal structures such as the statutes so that the processes for working on the content-related goals can then take place on this basis (Roloff 2008, Schuster 2023). The social interactions that occur within the processes also enable recurring patterns of behavior and thus informal structures. While, on one hand, network-specific institutions are established as a part of the ongoing processes, on the other hand, they provide the frame of action for the actors within processes taking place and create a certainty of expectation for the actors involved (Bathelt and Glückler 2018).

### Processes

Processes are procedures of change in an MSI that span over time. These are brought about by “[…] ways of communicating, sharing responsibility and taking decisions via instruments such as plans, committees and workshops” (Vangen et al. 2015: 1246). Consequently, formats in which actors interact, negotiate outcomes, or implement them are a relevant variable for conceptualizing processes. Formats formally established in the initiative’s constitution, such as general meetings, board meetings, and working groups, are just as possible as informal formats. Examples include telephone calls or personal conversations, which also contribute to how individual processes ultimately run. Functionally, the distinction can be related to the concepts of “formal bargaining” and “sense making” used by Ring and van de Ven (1994). Accordingly, it can be assumed that negotiations take place in formal formats, while in informal formats, in particular, positions are knocked down and trust is built up.

Negotiations in such formats are made possible by the existence of the specific internal institutions. Thus, the structuring network-specific institutions are the second relevant variable to capture processes. In the context of multisectoral collaborations, the importance of frequent interactions (Koch et al. 2023), trust (Getha-Taylor et al. 2019; Sloan and Oliver 2013) and power (Abarzúa and Glückler 2023; Levesque et al. 2017; Saffer et al. 2018; Schuster and Mossig 2022) has been studied. Analytically, different types of power in networks can be distinguished: Common categorizations are usually based on the source of the power capability. “Power over” on one hand is based on formally assigned decision-making powers, e.g., due to the position as an elected board member or the possession of relevant resources such as special financial resources. On the other hand, “power to” results from the power of persuasion and the ability to convince other actors of one’s own ideas and preferences (Allen 2003: 5–6; Mossig 2004). Recent work in the field of environmental politics has added “power with” to these two categories (Partzsch 2016: 6). “Power with” arises from learning processes of the actors involved, who question previous perceptions, have a strong transformational orientation, and actively strive for new forms of awareness of individuals or groups. They achieve substantial agency if they act in concert. The common perspective and an overarching solidary alliance as a source of power marks the central difference compared to “power to”, which usually refers to individuals or a single group and their actions (Partzsch 2016). It is therefore relevant to understand the forms of power that shape the processes within the network.

A third process dimension concerns instruments. In addition to instruments formally prescribed in the constitution, such as certain documents, reports and protocols (Schmidt 2017), informal instruments can also be used, such as agreeing on a certain procedure for decision making or the moderation of formats (Benighaus and Benighaus 2012), which guide the interaction between actors and thus support ongoing processes.

### Topics and Outcomes

Discussed topics and (not) achieved outcomes of collaboration in MSIs form a common element, as they are closely intertwined. The topics designate what the actors discuss in the formats. Expectations about topics can be derived from Glückler and Németh’s (2012) work on the governance of horizontal business networks. First, they point out that the rules of internal collaboration form a content of their own that needs to be clarified before actors can engage in solving the wicked problems. In addition to this procedural discussion item concerning the network itself, it is also obvious that the topics discussed is largely fed by the self-imposed objectives for solving the wicked problems that motivated the founding of the MSI in the first place.

Ideally, the topics discussed leads to concrete outcomes. In such collaborations, an essential outcome is that the actors involved make decisions about activities to be carried out (Vangen et al. 2015). Analogical to topics, Klijn et al. (2010) distinguish between procedural and substantive outcomes. The procedural outcomes concern networks’ internal courses of action. An example is represented by the self-determined constitution. The substantive outcomes are usually intended to contribute to the achievement of the MSI’s self-determined goals (Gray and Purdy 2018: 178).

The outlined theoretical framework for capturing the governance of MSIs based on the four elements of actors, structures, processes, and topics and outcomes is to be used in accordance with the research question posed in this paper in order to explain (un)achieved outcomes of the MSI studied. For this purpose, four sub-questions can be derived from the explanations above, which address the four elements of governance and will be answered using the case study of the “German Initiative on Sustainable Cocoa”:

1. Why do which actors participate in the MSI?
2. What features characterize the structures of MSI?
3. How do processes work in the MSI?
4. What topics are discussed, what outcomes are (not) achieved?

## The “German Initiative on Sustainable Cocoa” Case Study

The German MSI “*Forum Nachhaltiger Kakao*” (German Initiative on Sustainable Cocoa [GISCO]), which was registered in April 2014 as an association based in Berlin, serves as a case study (Forum Nachhaltiger Kakao 2022; Forum Nachhaltiger Kakao n.d.): 70 organizations are engaged in it as members from the German cocoa and chocolate sector. Each actor belongs to one of the four actor groups:

- The German public sector, represented by the Federal Ministry for Economic Cooperation and Development (BMZ) and the Federal Ministry of Food and Agriculture (BMEL);
- the German cocoa, chocolate and confectionery industry with 44 organizations;
- the German food trade with seven organizations and
- civil society with 17 organizations.

Each of the four stakeholder groups sends two people to the MSIs Executive Board, which has a total of eight members. In addition, GISCO has a Secretariat and thus represents an MSI that is coordinated as an NAO (described in section 2).

The members of GISCO have agreed on three overarching goals: first, to improve the living conditions of cocoa farmers and their families by contributing to a secure livelihood; second, to conserve and protect natural resources and biodiversity in the cocoa producing countries; and third, to increase the share of sustainably produced cocoa and its marketing (Forum Nachhaltiger Kakao 2019). To this end, representatives of member organizations can actively participate in GISCO by attending the annual general meetings and getting involved in one of three working groups (WGs). The three WGs are responsible for (1) public relations, (2) outlining further measures to increase the sustainability of cocoa on the German market, and (3) monitoring the “PRO-PLANTEURS” project. In the latter, the focus is on improving the socioeconomic living and working conditions of 30,000 farmers in Côte d’Ivoire (Forum Nachhaltiger Kakao 2020). GISCO thus corresponds to the “continuous improvement model” (Buckley et al. 2019) of MSI described in section 2.

## Methodology

Two methods were used to capture GISCO’s network governance empirically. First, the minutes of 84 committee meetings from May 2014 to the end of December 2019 were analyzed. The minutes of the general meetings, board meetings, and working group meetings were provided exclusively for analysis. The minutes contain three pieces of information relevant to the analysis: First, they record which actors were present at the meetings. Second, the minutes provide information on what was discussed in the meetings and what decisions were made. And third, the minutes indicate which actors in GISCO were given tasks. This makes it possible to count the delegations recorded in the minutes and plot them on a network graph, so that it becomes clear whether the actual interaction in GISCO corresponds to the formally defined structures (Schuster 2023). To this end, each listing of a person, organization or committee responsible for a task (hereafter referred to as delegation) in the 84 minutes was counted and added up over the entire study period. These may be actors within or outside the network, as board and working group meetings do not only delegate responsibilities to members or committees of GISCO. Therefore, only those actors who have either delegated or received responsibilities are part of the network.

The delegations can be understood as ties between sending and receiving actors (or nodes). Since the senders, receivers and the number of ties or delegations are known, in-degrees and out-degrees can be calculated and displayed in the form of network graphs. The indegree of a node indicates how many ties (delegations) are received from other nodes, i.e., how many delegations this node receives from board and working group meetings. Conversely, the outdegree indicates from which nodes a particularly large number of ties to other nodes are sent (Fuhse 2018: 57–60), i.e., which of the committees delegate the most responsibilities. Central results of the protocol analysis are described in section “Empirical Findings Regarding Structures”.

However, the protocols lack the reasons underlying arguments or concessions upon which the respective decisions were reached. This gap was closed with the help of guided expert interviews (Bogner et al. 2014). For this purpose, a guide was drafted (Kaiser 2014: 55–70), containing questions about governance based on GISCO’s conceptual framework. The guide was evaluated together with a person from the Secretariat and constantly refined between interviews. Fifteen individuals representing member organizations were interviewed from April to August 2020. The sample includes at least one person from each stakeholder group. In addition to the 15 interviews, three supplemental interviews were conducted. One interview was conducted with two staff members from the Secretariat. Two additional interviews were conducted with a representative of a member organization and a representative of the Secretariat while preparing the research project in 2018. The evaluation is thus based on a total of 18 interviews. With one exception, all the experts are or were involved in the Executive Board or in working groups and are therefore likely to have a strong knowledge of governance-relevant processes in GISCO.

Due to COVID-19, interviews were conducted by telephone or video call in the summer of 2020. This limited the quality of the interviews only slightly but led to a challenging interview process as described by Christmann (2009). The average duration of the interviews was 65 min. All conversations were recorded with a voice recorder and transcribed into written German using easily accessible transcription rules following Kuckartz (2018: 166–168). All interview partners authorized the transcripts after the interview. Each interviewee was assigned a unique abbreviation consisting of a letter and a number. The letters “A” to “D” stand for membership in one of the four actor groups, “G” for the Secretariat. The numbering is applied continuously and has no meaning in terms of content. Subsequently, a qualitative content analysis (Kuckartz 2018: 97–122) was conducted using the qualitative data analysis software MAXQDA. During the first step, around 650 text passages were marked as relevant in all 18 transcripts and around 500 memos were written. In the following process, the main categories “actors,” “structures,” “processes,” and “topics and outcomes” derived from theory are then detailed through subcategories, formed inductively from the transcribed interview material. Seven steps were carried out in accordance with Kuckartz (2018). This resulted in 2.364 codings in 135 categories organized in five hierarchical levels.

## Results

The presentation of the results corresponds to the elements of the theoretical framework. First, the actors and their motives for participating are presented (section “Empirical Findings Regarding the Actors”). The results regarding the structure are based on the protocol analysis and are supplemented by the expert interviews (section “Empirical Findings Regarding Structures”). The design of the processes taking place in GISCO are outlined based on the interviews (section “Empirical Expression of the Element of Processes”). Finally, the topics and outcomes that were (not) achieved from the experts’ point of view are presented (section “Empirical Expression of Topics and Outcomes”).

### Empirical Findings Regarding the Actors

In the theoretical framework, three relevant aspects regarding the governance element of actors were highlighted: First, the composition of the actors and a possible “invisibilization effect” (Bitzer and Marazzi 2021: 382) due to non-participating actors; second, whether or to what extent the actors differ with regard to their motivation to participate; and third, the identification of particularly influential actors.

No indications emerged from the interviews that important actors in the cocoa and chocolate sector in Germany or relevant civil society groups concerned with this area are missing. The actors represented cover the main positions within Germany. However, in view of GISCO’s three central goals (improving the living conditions of cocoa farmers, conserving natural resources in the cocoa producing countries and increasing the share of sustainably produced cocoa), it is questionable whether a purely German composition of the actors is a reasonable choice. There is a lack of voting members from the Global South, both on the part of the cocoa producers as well as the governments and civil society there. As the relevant actors are members from Germany, an “invisibilization effect” (Bitzer and Marazzi 2021: 382) can certainly be identified on a global scale. The goals formulated by GISCO directly affect labor law, social legislation and environmental law in the respective cocoa producing countries. The experts interviewed stated very clearly that the company’s own “PRO-PLANTEURS” project can serve as a role model in this regard, but nothing more. Thus, the lack of involvement of relevant actors from the Global South is already a central explanation for (not) achieving globally far-reaching outcomes.

**Fig. 1:**
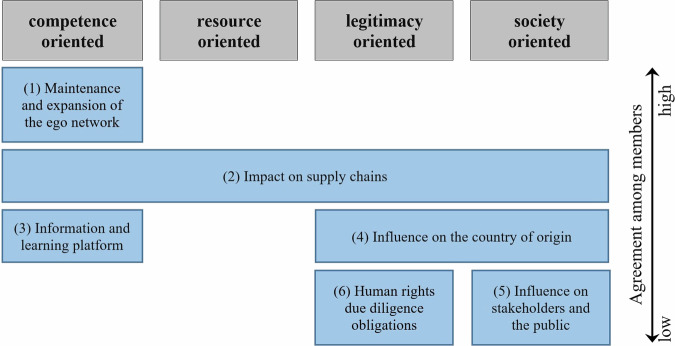
Functions of GISCO for member organizations and degree of overlap between the groups of stakeholders (own illustration)

According to Gray and Purdy (2018: 27–34) the reasons for engaging in an MSI can be distinguished into competence-, resource-, legitimacy-, and society-oriented participation motives. The experts were therefore asked in the interviews about the added value of participation for their organization. From this emerged six functions the MSI serves for its members which can be assigned to the four participation motives mentioned in the theory (Fig. 1).

It is clear from the interviews that the motives for participating in GISCO vary, both between and within groups of actors. A high degree of agreement exists (1) in the opportunity for actors to maintain and expand their ego networks in GISCO. By grouping relevant actors in the cocoa and chocolate sector, it is possible to approach important people and get to know them without much effort. This feature is mentioned by experts from all stakeholder groups. Companies use the MSI to meet and exchange ideas with actors relevant to them, such as competitors, customers, politicians or representatives of civil society. The representatives of civil society also emphasize the possibility of gaining access to contacts in politics and business as well as potential partners for joint projects through membership. It is therefore clear that actors have their own interests associated with the membership (Grabs and Ponte 2019, Søreide and Truex 2013).

(2) Some members of various stakeholder groups also share the endeavor to influence their (own) supply chains through membership in GISCO. This is linked to different motivations. It is not only about the procurement of cocoa as a central resource, but also about the development of one’s own competencies regarding societal aspects and thus one’s own legitimacy as the quote from company representative B2 (2020: para. 111) illustrates: “Our learning process is mainly focused on how we can improve our supply chain, in terms of human and environmental rights”.

Less overlap can be seen in the following two functions of the MSI for member organizations, which are named by representatives of different actor groups but interpreted differently. This illustrates that the differences in the prioritization of motives described in the literature can also be found in the case of GISCO (Grabs and Ponte 2019, Gray and Purdy 2018: 27–34): In the case of GISCO’s function as (3) an information and learning platform, actors partly want to tap into different forms of knowledge. A broad majority mentions learning from and about other actors and groups of actors as well as obtaining information and development trends in the cocoa and chocolate sector that would otherwise be more costly, occur later or would not be accessible at all. Civil society representatives, on the other hand, would like to better understand how companies operate and illuminate experiences with the potential and limitations of MSIs as a way to achieve sustainability goals.

Many stakeholders would like to (4) influence the sustainability of the cocoa and chocolate sector, respectively the socio-economic conditions of the farmers in the cocoa producing areas. Many industry representatives who mention this goal link the exertion of influence to the GISCO project “PRO-PLANTEURS.” In contrast, respondents from civil society pursue the goal of influence more comprehensively and independently of the project. Thus, the most important goal for D1 (2020: para. 49) is “[…] that one could really speak of sustainable cocoa. That there is no more child labor, that there is no more poverty among cocoa farmers.” This statement is in line with Gray and Purdy (2018) claim that civil society organizations demonstrate society-oriented participation motives.

Two further functions of GISCO for its member organizations can be identified, which, however, do not seem to be equally important to all actors and therefore show a low degree of overlap between the actor groups. This is the case for (5) targeted influencing of individual stakeholder groups, such as policy makers, and public discourse on sustainability in the sector. In this regard, some respondents even described their impressions that in GISCO individual actors would try to block progress in general and a supply chain law in particular. For two representatives of large companies, membership also serves the function of indirectly contributing to the (6) fulfillment of human rights due diligence obligations.

If the participation motives mentioned are contrasted with the self-imposed goals of GISCO, it becomes apparent that the desired ecological and social improvements in the cocoa producing countries of the Global South represent a rather subordinate motive of the actors surveyed for participating in the MSI, and this results in a further indication for explaining (non-)achieved outcomes: The lack of overlap between participation motives and the intended goals of the MSI.

The theoretical framework suggests identifying the most influential actors possible. Which actors or which groups of actors are particularly influential will become apparent during the analysis of the structures and processes.

### Empirical Findings Regarding Structures

**Fig. 2:**
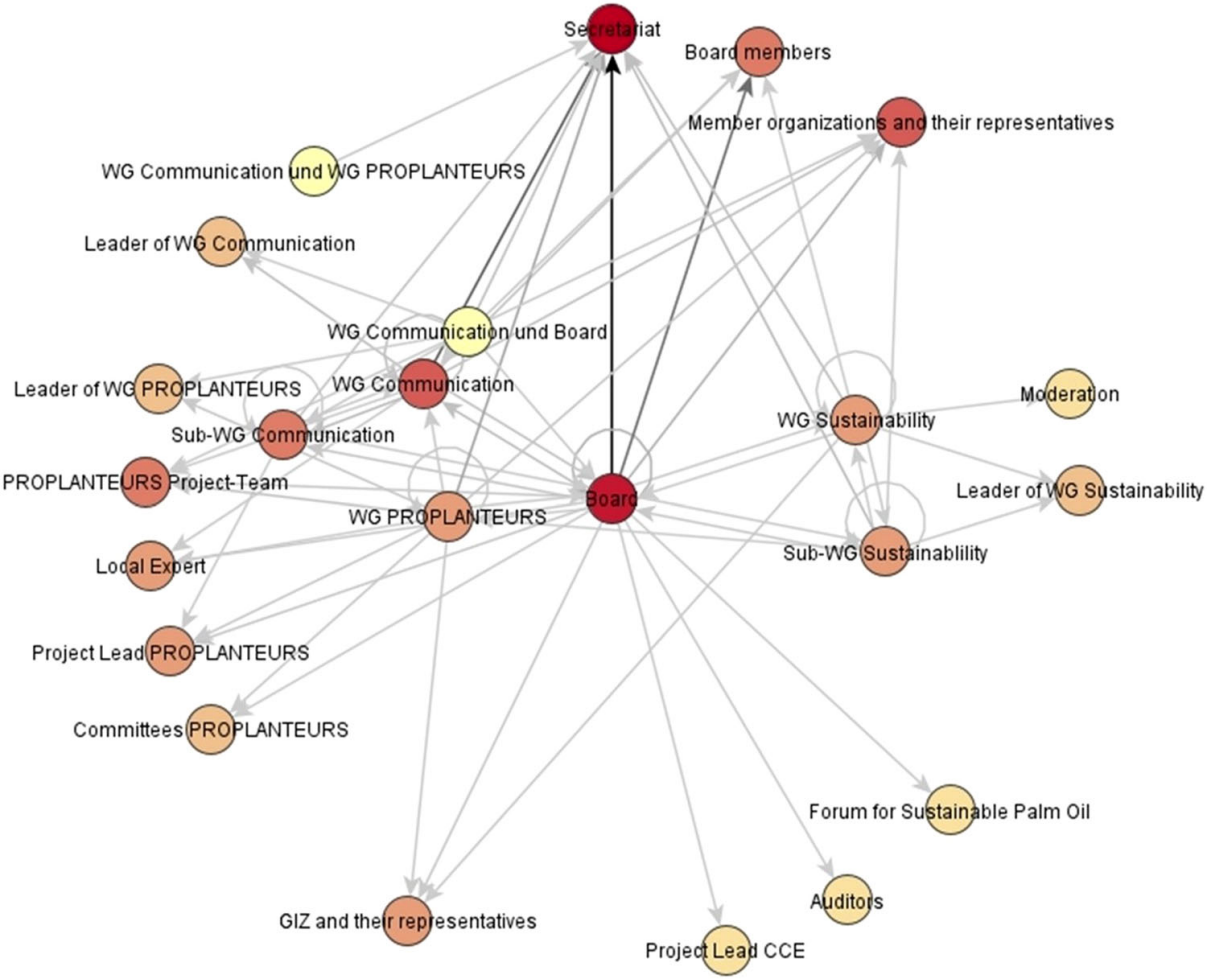
Network of delegated responsibilities (own illustration)

The structure of the MSI as a constituent element of network governance (Provan and Kenis 2008) can be represented in the form of an organizational chart. On the other hand, the theoretical framework explicitly considered informal network structures, which may differ from the definitions in the association’s constitution (Glückler and Németh 2012; Abarzúa and Glückler 2023). The protocol analysis allows for a comparison by creating a network graph from the count and summary of the actual delegated responsibilities in the committee meetings using the VISONE software (see Fig. 2). This means that it is not the structure of the network itself that is examined, but the extent to which the formal structure of GISCO is actually used by the committees (Schuster 2023). The network created in this way consists of 23 actors and committees from inside and outside GISCO, which are connected to each other as nodes by a total of 67 edges. Shown are the centrality measures of the outdegree and the indegree as well as the edge strength.

In terms of GISCO, a high number of connection partners is interpreted as an influence on network events. Because the delegating committee and the respective recipients are known each time a task is delegated, the relationships are directed. Committees and individuals that send or receive responsibilities are defined as nodes in the network. They are connected to each other through delegation of responsibilities, which are the edges of the network. Consequently, it can be calculated and represented as an outdegree which nodes (committees) emanate particularly many edges (delegations) to other nodes (Fuhse 2018: 57–60). It is important to note that committees can also delegate responsibilities to actors (nodes) who are not members of GISCO. For example, at one board meeting, external organizations were asked to provide GISCO with additional information.

In Fig. 2, the outdegree determines the distance of the node from the center of the network. The Executive Board is located there as the node with the highest outdegree in the network, because about 25 percent of the total 67 edges in the network emanate from it. This means that the Executive Board, as part of the NAO, reaches most actors and committees directly and can coordinate cooperation. The peripherally positioned actors (e.g., Secretariat, local experts, moderators of the sustainability WG) have an outdegree of zero, as no delegations of responsibility were recorded from them.

Indegree can be used to calculate which nodes (committees and people) receive a particularly large number of edges (delegations) from other nodes (committees) (Fuhse 2018: 57–60). The darker a node’s color in Fig. 2, the more edges run to it or the more is delegated to it. Twelve percent of all edges in the network run to the Secretariat, which means it has the highest indegree of all nodes. It thus functions as the executive unit of the NAO, to which many committees delegate responsibilities. In addition, the Executive Board also has a high indegree of eleven percent of all incoming edges. As a higher-level body, the Executive Board is involved by the working groups through delegations when appropriate, e.g., for decisions.

The third network parameter visualized was the edge strength in the network graph, which reflects the frequency of interactions. The darker an arrow’s color, the stronger the relationship between two committees. About one-third of the total of approximately 1500 recorded delegations during the study period from May 2014 to December 2019 are related to the connection from the Executive Board to the Secretariat. The working groups also have intensive relationships with the Secretariat, to which they delegate a particularly substantial number of responsibilities.

The analysis of the delegation of responsibilities confirms that the division of functions between the different bodies corresponds to the formally defined structures in the organizational chart. Thus, GISCO corresponds to an NAO in its pure form, as described by Provan and Kenis (2008), and a NAO appears suitable for such a complex network with a relatively large number of participants.

The expert interviews confirm the “lived structure” of GISCO. However, although the interviews emphasized the importance of individual people, that is not evident from the organization chart or the network graph. This confirms the assumption from the literature that individuals influence the actions of a partnership (Vangen et al. 2015) especially if these frequently engage in reciprocal communicative exchange with different actors and thus overcome existing boundaries (Koch et al. 2023). On the one hand, this relates to the role of the board members, who, as representatives of their respective stakeholder groups, represent common positions on the Executive Board. On the other hand, the eight board representatives connect their actor groups to the Executive Board by communicating with their groups before and after board meetings, sharing information, reporting on activities, in some cases advocating for agreement on compromises, pre-discussing votes, and bringing back decisions from the board meeting to the respective internal meetings of the actor groups. Still, people also have the opportunity to influence cooperation through competent action, an appropriate personal appearance and perseverance, without holding office. The experts’ descriptions gave no reason to assume that such informally legitimized individuals would override the formal structures of collaboration through their influence in the committee meetings. In contrast to the work of Abarzúa and Glückler (2023), GISCO corresponds to the formally planned structure and is not overlaid by an informally created network. Overall, the (un)achieved outcomes indicate that formal and informal structures correspond and that there are therefore no structural difficulties in decision-making processes.

### Empirical Expression of the Element of Processes

Following Vangen et al. (2015) processes are shaped by formats, institutions, and instruments. It is noteworthy that the constitution and regulations of the association as formal institutions (North 1990) do not contain any specifications as to how exactly processes in GISCO must be designed. Similarly, the conduct of members is not governed by a code or the like, and the constitution does not include formal arbitration. The constitution merely provides a regulatory framework that specifies how and in which committee decisions are made. Nevertheless, the process flow is described by the interviewed experts as a now well-rehearsed procedure of discussing and negotiating topics in certain formats and in an established sequence.

As a rule, new topics are first discussed by the Executive Board and, if necessary, delegated for further elaboration to one of the three WGs: Communication, PRO-PLANTEURS or Sustainability. In these WGs, the efforts of the voluntarily present members to achieve outcomes in line with the self-imposed goals are comparatively great. Likewise, the influence of individual people still seems to be relatively great at this stage. According to Allen (2003) and Mossig (2004), people with a high capacity of *power to* have the opportunity to use it at this point. If a recommendation to the Executive Board is agreed upon within the WGs, the resolution prepared by the Secretariat is distributed to the individual stakeholder groups via the board members. The development of a common position within the stakeholder groups serves to prepare the board meetings. The evaluation of the resolution by the actor groups can sometimes deviate quite strongly because the actors within their group may well have different positions. “Someone makes a proposal, and then you look at it, and it sounds good at first, and then it always goes back to the member groups again, and you notice that it then becomes much more cumbersome” (B5 2020: para. 35). It is clear from the interviews that the influence of individual people in support of the proposal in this process step is recognizably smaller than previously in the WGs. Therefore, formal *power over* (Allen 2003, Mossig 2004) in the form of veto rights is significantly greater than the *power to* of individuals at this stage of the process.

After the four stakeholder groups have commented on the recommended course of action, the board members convene in a board meeting to make a decision. When GISCO was established, the people involved agreed that none of the four stakeholder groups involved can be outvoted on board decisions. If there is no consensus among the stakeholder groups, the board representatives discuss these aspects and try to find a compromise. One person from the Secretariat perceives it as very helpful if the board members of different actor groups also exchange ideas between meetings and discuss their respective positions with each other, which corresponds to the distinction outlined by Ring and van de Ven (1994) between formal negotiations in official bodies and “sense-making” outside of them. If a compromise cannot be reached in the board meeting, the discussions are delegated again to the WGs and/or discussed again in the individual actor groups. This loop is repeated with varying frequency, depending on the topic, until either a consensus is reached among the stakeholder groups, or the topic is taken off the agenda and not pursued further.

Overall, it becomes apparent that civil society members can use their veto power as a special form of *power over* to a lesser extent than their company counterparts. Civil society actors rely on arguing, mobilizing other groups of actors for their own goals, and thus try to use their *power to*. This confirms research that suggests power resources are usually unequally distributed in such networks (Abarzúa and Glückler 2023). At the same time, this example shows that power with (Partzsch 2016) is not very prevalent within GISCO. Despite the agreement on ambitious goals, the willingness to learn and rethink previous perceptions does not seem to be profoundly developed among many stakeholders as to result in substantial agency. In particular, it has not been possible to create a common perspective between the stakeholder groups that would allow them to unite in solidarity and use this power to achieve the desired transformations in the cocoa and chocolate sector. Regarding GISCO’s self-imposed goals, it should be noted that compromises reached are often the lowest common denominator between the actors. Interviewee G2 formulates the struggle for outcomes as follows: “And we often find consensus and it is always compromise. It is very rarely the case that there is a proposal and then everybody goes along with it.” (G2 2020: para. 87). The fact that compromises reached in the working groups were withdrawn again by individual groups of actors in the Executive Board was seen by some interviewees as a deliberate strategic tool. It is also apparent that the stakeholder groups are in a strategically more favorable position than the WGs, because they can again improve agreements reached in the WGs according to their interests or prevent them altogether. Thus, the specific process flows in GISCO can be seen as another reason its self-imposed goals are achieved only hesitantly. In particular, the agreed unanimity and the resulting veto possibilities prove to be obstacles in the process flows.

### Empirical Expression of Topics and Outcomes

During the protocol analysis, discussed topics and decided outcomes became visible. Based on this, the experts were asked in the interviews about what they considered to be the most relevant outcomes of the collaboration. The analysis of the answers draws on the distinction made in the theoretical framework between process-related and content-related outcomes (Klijn et al. 2010). The outcomes described vary in their (in)direct effects on the intended sustainability goals of GISCO.

There are some outcomes that are achieved as a result of the continuous action and interaction of the actors in GISCO. The very existence of GISCO as an industry-specific exchange platform and the knowledge acquired there as well as the discussion culture, which is perceived as objective from the experts’ point of view, is named as an outcome achieved. Some of the interviewees also consider the associated holding of substantive discussions on the objectives of the association, such as living wages or the Supply Chain Act, to be an outcome that has already been achieved. They point to a certain radiating power and a growing awareness through the establishment of the MSI itself, because already the exchange about topics can “[…] bring a lot of small outcomes, which you cannot measure at all. But they are incorporated somewhere in everyone’s work” (B2 2020: para. 117). These findings are consistent with Glückler and Németh’s (2012) observation that horizontal business networks of actors first address its own needs before achieving further results.

In addition, there are outcomes on the network level. However, they are specifically sustainable as decisions taken by the association bodies. In the interviews, the development of the GISCO’s own definition of sustainable cocoa, the formulation of recommendations for action for the individual groups of actors, and the revision of goals including the creation of a monitoring system, with which civil society actors hope to motivate companies through the publication of information to “really do more for sustainability than just buy certified” (D3 2020: para. 51), were repeatedly mentioned as important resolutions. In particular, the establishment of the Sustainability WG, from which many strategically relevant resolutions originate, is highlighted in this regard. However, D2 (2020: para. 62) reminds of the necessity to achieve outcomes in the cocoa producing regions: “With the WG, we have successfully managed to initiate the exchange of content between the actors. But that alone does not bring anything to the farmers on the ground. And that is where we as a civil society organization think about how much time we invest if it does not have an impact on the ground.” Thus, the achieved outcomes confirm the assumption of Vangen et al. (2015) that an essential outcome of a network is that the actors involved make decisions about activities to be carried out. Those activities contribute to the achievement of the MSI’s self-defined goals as Gray and Purdy (2018) stated.

The “PRO-PLANTEURS” project in Côte d’Ivoire is assigned as an outcome which relates directly to the targeted objectives regarding the cocoa and chocolate sector and thus has a higher reach than the outcomes mentioned so far. However, GISCO’s own project is limited only to selected cocoa producing regions of Côte d’Ivoire. While for some interviewees “PRO-PLANTEURS” is a “great outcome of the forum” (C1 2020: para. 41) or “one of the most important things […] we have touched and are implementing” (B7 2020: para. 119), it seems less relevant for other stakeholders. The project is considered to have comparatively little direct impact in terms of solving challenges across the cocoa and chocolate sector due to its limited budget and narrow geographical focus. However, knowledge gained could directly and indirectly feed into the work of GISCO and stakeholders involved. Accordingly, the impact of “PRO-PLANTEURS” on GISCO and the cooperation of the actors within it is named as a relevant outcome of the MSI.

Going beyond “PRO-PLANTEURS” and take the entire cocoa and chocolate sector as a reference point of outcomes, the experts assess the direct effects of GISCO on the sector as low and refer to the need for legal regulations. It is true that reference is made to the steadily increasing proportion of certified cocoa processed by GISCO member companies for products on the German market. However, representatives of all stakeholder groups are aware that certifications in the cocoa producing regions do not necessarily lead to better living and working conditions for the farmers. Indirectly, however, many experts indicate that GISCO makes a positive contribution to the further development of the sector, among other things through cooperation with international initiatives.

However, the self-defined goals of GISCO are formulated in such a way that with the intended improvement of the living and working conditions of the cocoa farmers and their families, outcomes beyond the network level are aimed for. The analysis of the statements shows that the interviewed actors are aware, however, that their contribution to this as an MSI is at most indirect and that they cannot achieve these goals on their own. Instead, their direct influence possibilities do not exceed their own project. The positions of the different stakeholders involved are too heterogeneous to achieve results (so far) that have an impact on a global scale. In a MSI that corresponds to the “continuous improvement model” (Buckley et al. 2019) long-term developments, disagreements among actors and the absence of any legally binding effects (Mena and Palazzo 2012) are a part of business. Additionally, the situation is complicated by the fact that key shareholders from the Global South are not represented in GISCO, which is described by Bitzer and Marazzi (2021) as an “invisibilization effect”. The MSI must therefore accept criticism from outsiders that they promise more in their goals than they can actually achieve through a direct contribution.

## Conclusion

Based on the conceptualization of governance of an MSI according to the continuous improvement model (Buckley et al. 2019), four questions were derived, from which the empirical results were presented: (1) Why do which actors participate in the MSI; (2) what features characterize the structures of the MSI; (3) how do processes in the MSI run and what topics are discussed; (4) what outcomes are (not) achieved?

The elements of the theoretical framework addressed by these questions have been shown to be suitable for empirically capturing the governance of an MSI. Thus, the presented framework contributes to filling the research gap by developing a theoretical basis that had been missing (Abarzúa and Glückler 2023; Bryson et al. 2015; de Bakker et al. 2019; Gazley 2017; Getha-Taylor et al. 2019; van Tulder et al. 2016) and to which future empirical studies on the functioning of MSIs can now refer.

Conclusions and implications can be drawn along the four elements of the theoretical framework. With regard to the *actors*, the target groups of the self-imposed goals must be included directly and on an equal level in order to avoid “invisibilization effects” (Bitzer and Marazzi 2021). No actors from the Global South are represented in GISCO, although the central objectives address the improvement of the living conditions of cocoa farmers and their families in the producing countries. Representation of the target groups would have the advantage that planned activities and programmes could be better contextualized and tailored to local needs—beyond the cooperation in the “PRO-PLANTEURS” project in Côte d’Ivoire. Furthermore, the purely national composition of GISCO must be questioned as global economic relationships can only rarely be shaped by activities at the national level.

In terms of *structures*, the study shows that no informal parallel structures have emerged, as has been observed in other MSIs (Abarzúa & Glückler 2023). In this respect, the organizational form as a NAO (Provan and Kenis 2008) with a permanent secretariat has evidently proven its worth. However, the analysis of the *processes* shows that the lack of hierarchical directives, sanctions and other decision-making mechanisms makes negotiation-based compromise difficult in the MSI studied. Consequently, the power of individual groups of actors to use their veto right to significantly delay or completely prevent the changes required to achieve goals cannot be overcome. Ultimately, the division between the groups of actors into “us versus them” (Koch 2024) continues to exist and no common narrative has emerged to generate power with (Partzsch 2016). It could be advisable to avoid the loop through the stakeholder groups after finding a compromise within the working groups in order to strengthen the working groups and the Executive Board. In consequence, veto players would have to express their opposing positions much earlier within the working groups. Based on the findings of Koch et al. (2023), it would be highly advisable in particular for the board members as central protagonists, to communicate intensively with other stakeholders from all of the different stakeholder groups that do not belong to the board. This means that they should see themselves less as representatives of the interests of their own stakeholder group and more as representatives of the jointly pursued goals.

With regard to the *goals*, it should be noted that more achievable goals should be addressed. GISCO’s goals are very ambitious and can be communicated well to the public due to their general relevance. However, the achievement of these goals is mostly beyond the MSI’s own sphere of influence and therefore nearly only outcomes on a network level and within the own project “PRO-PLANTEURS” were accomplished.

At the same time, the study shows the potential of MSI as a learning and networking platform. Thus, an MSI based on the continuous improvement model can contribute to compensating for a lack of legal regulations through the consensus of competitors. This can be seen, for example, in the steadily increasing share of certified cocoa and the market coverage of GISCO members, who together represent 88 percent of the cocoa volume from cocoa-containing end products on the German market (Forum Nachhaltiger Kakao 2022).

Although the context in environmental governance is different, the results show parallels to the study by Siangulube (2024), which examines how stakeholder groups perceive the role of MSIs in addressing landscape challenges. GISCO’s outcomes correspond more or less to the first two narratives he found using the example of stakeholder groups in Zambia’s Kalomo District. According to this, MSIs represent (1) institutions that foster dialog and (2) support market-driven solutions to solve problems. The third narrative found by Siangulube (2024) recognizes power imbalances and considers MSIs as institutions to identify policy gaps and needs. GISCO does not fulfill this role adequately, as the unachieved outcomes on a global scale show.

Instead of analyzing the meeting minutes further research could capture communicative interactions of the actors and examine them as a network. This would allow to draw on the finding by Koch et al. (2023) that the frequency of interactions and trusted leaders with many reciprocal connections to different actors, are an important prerequisite for a common narrative to emerge in order to overcome existing boundaries between the actor groups. This could also identify existing conflicts that were not revealed in the protocol analysis but are very likely to be a relevant reason why sustainability goals are not achieved. To this end, a network survey as suggested by Glückler et al. (2020) could be carried out with interviews with all stakeholders of an MSI.
